# Management options for vulvar carcinoma in a low resource setting

**DOI:** 10.1186/1477-7819-8-94

**Published:** 2010-11-01

**Authors:** Ahizechukwu C Eke, Lilian I Alabi-Isama, Josephat C Akabuike

**Affiliations:** 1 Department of Obstetrics and Gynecology, Nnamdi Azikiwe University Teaching Hospital, Nnewi, Anambra State, Nigeria; 2 Division of Surgery, Oncology, Reproductive Biology and Anesthetics, Imperial College, Hammersmith Campus, Du cane Road, London W120NN, UK

## Abstract

**Background:**

Vulvar carcinoma is a rare tumor of the female genital tract. In Nigeria, very few studies have looked at the management options for vulvar carcinoma. The objective of this study was therefore, to describe the management options available and the challenges in treating this malignancy in Nigeria.

**Methods:**

A descriptive study of all vulvar cancer cases managed at the Nnamdi Azikiwe University Teaching Hospital, Nnewi over a 12 year period (1998-2009). The theatre, ward register, histo-pathologic records and case notes of all women who had surgery for vulvar carcinomas were retrieved and socio-demographic characteristics, clinical presentation, type of surgery, histologic type and complications of treatment were retrieved and analyzed.

**Results:**

There were 867 gynecological malignancies and vulval carcinoma accounted for 11 cases, giving a prevalence of 1.27%. The ages ranged from 54 to 79 years with a mean of 61.2 years. Parity was 2-14, with a mean of 6.7± 2.33. Most of the patients were of low socio-economic class. All the 11 patients had surgery as 1^st ^line treatment. Radical vulvectomy was done for 6 cases since they presented in the advanced stage. The complications of surgery included hemorrhage (18.2%), chronic lymphedema, wound infection and anesthetic complications. There were no hospital mortalities. Late presentation, with stage III (45.4%) was the commonest stage at presentation while the majority of the vulvar carcinomas (72.7%) were of epithelial origin. Squamous cell carcinoma predominated (63.6%).

**Conclusion:**

Carcinoma of the vulva is a rare gynecological malignancy in Nigeria. Surgery and radiotherapy remains the mainstay of this disease in Nigeria and can be highly successful if patients present early.

## Background

Vulvar cancer is one of the rarest gynecological cancers in Nigeria [1]. In a study in Zaria, Nigeria, it accounted for 1.2% of all genital tract malignancies [1]. It affects 3 in 100,000 women per year and accounts for 4% of female genital tract malignancies in the United Kingdom [2]. It is a disease of older women, with over 80% of cases occurring in women over 55 years old (between the ages of 60-70) [2,3]. Up to 15% of vulvar cancers are diagnosed in women under 40 years old [2,3]. Possibly due to human papilloma virus infections, clinicians are treating increasing numbers of younger women, which presents unique challenges in managing this cancer [1-3].

Carcinoma of the vulva may arise from the skin, subcutaneous tissue, glandular elements of the vulva or the epithelium of the lower third of the vagina [4]. Of all the vulval carcinomas, 1-2% are basal cell carcinomas, with the majority being squamous cell carcinomas (90%) [2,4]. Less common primary cancers are malignant melanomas, carcinoma of Bartholin's gland, sarcomas, Paget's disease of the vulva and sweat gland cancers [4,5]. Although vulvar carcinoma is more common in the elderly in most parts of the world, the incidence is rising in all age groups [4]. A study done in Nigeria 20 years ago showed that vulvar carcinoma was commoner among women of low socio-economic status [6]. It is primarily a disease of post-menopausal women, with peak incidence in women aged 60-70 years [4,6].

Despite the advanced age of many of these patients and the frequent finding of a moderately large tumor, the disease is usually amenable to surgical and radiation therapy. In stages I and II disease, the corrected 5 year survival rate is greater than 90% [1,4].

The traditional surgery for vulvar carcinoma has been radical vulvectomy with superficial and deep inguinal node dissection through a single incision with at least 2-cm margins around the tumor [4]. Treatment of advanced cases of vulvar cancer is usually followed by adjuvant radiotherapy for most patients [4]. Surgical approaches in the treatment of vulvar cancer have changed significantly over time because of the significant peri-operative morbidity and mortality from radical vulvar surgeries. More conservative operative approaches are used today that have fewer complications and are less disfiguring than the radical vulvectomy and bilateral inguinal node dissection commonly practiced in the past. These conservative approaches include unilateral inguinal node dissection for small ipsilateral tumors, the triple-incision technique, wide local excision with 1-cm margins, and saphenous vein sparing surgeries, with attempts made to prevent lymphedema [4].

The objective of this study was to describe the management options for vulvar carcinoma available in Nigeria, and the challenges encountered in the management of these vulvar malignancies.

## Methods

This was a descriptive study of all cases of vulvar carcinoma that presented to the Nnamdi Azikiwe University Teaching Hospital (NAUTH), Nnewi, South-east, Nigeria, between 1^st ^of January, 1998 and 31^st ^of December, 2009 (a 12 - year period). The case files of 11 women that presented with vulvar carcinoma and were managed at the Nnamdi Azikiwe University Teaching Hospital, Nnewi from the 1st of January, 1998 to the 31^st ^of December, 2009 were retrieved and studied retrospectively. Thorough scrutinies of the gynecology ward and theatre unit records were done to identify these patients. The total number of gynecological malignancies over the same period was also obtained. Also, the total number of women that presented with gynecological problems during the study period was determined.

Ethical clearance was obtained from the ethical committee of the Nnamdi Azikiwe University Teaching Hospital, Nnewi, Nigeria prior to the collection of the case notes. These case-records were studied and variables such as age, parity, clinical presentation, stage of disease, type of surgery, histologic type of vulvar carcinoma and complications of treatment were collected on a data extraction form and were subsequently analyzed. Where a patient had surgery, the above information was confirmed by comparison with information in the operating theatre record books. All the patients had histologically proven vulvar carcinoma, though details of histological diagnosis were not included in this study. Epi-info version 3.3.2 was used in computing the data into percentages. A p-value of <0.05 at 95% confidence interval was taken as significant.

## Results

During the period of study, there were 11 women admitted into the gynecological unit with vulvar carcinomas. There were a total of 867 gynecological cancers over the 10 year study period. The total number of patients admitted to the gynecological unit during the same period was 3,418. Thus 25.4% of the gynecologic admissions were for gynecological cancers and 1.27% of the gynecological cancers were vulvar carcinomas. The largest numbers of vulvar cancers occurred in the fifth to seventh decades of life; 82.7% of the cancers occurred in these age groups. The ages of patients studied ranged from 54 to 79 years and the mean age for vulvar carcinoma was 61.2 years. The parity of the women treated ranged from 2-14. The mean parity of the women treated was 6.7± 2.33. Most of the patients were of low socio-economic class.

### Clinical presentation (Table 1)

**Table 1:** The Presenting symptoms of patients with vulvar carcinoma

*Symptom*	*Frequency*	*Percentage (%)*
Pruritus vulvae	9	26.4
Vulvar swelling/lump	5	14.7
Vulvar discharge	5	14.7
Vulvar pain	6	17.8
Urinary tract symptoms	2	5.9
Vulvar bleeding	7	20.5

**Total**	**34**	**100.0%**

*Some patients presented with more than one symptom.

All the patients that presented with vulvar carcinoma presented with symptoms. Some patients presented with more than one symptom. Nine (26.4%) of the patients presented with pruritus vulvae, while 5(14.7%) of the patients presented with vulvar swelling/vulvar mass.

### Histological type of vulvar carcinoma

The commonest histological type of vulvar carcinoma noted among the 11 patients that presented with vulvar carcinoma was squamous cell carcinoma, seen in 7(63.6%) of the patients. There was one (9.1%) case of Bartholin's gland adenocarcinoma and one case (9.1%) of basal cell carcinoma of the vulva. A case (9.1%) of Paget's disease of the vulva and 1 case of a sarcoma of the vulva were also noted. No cases of verrucous carcinoma, melanoma, lymphomas or metastatic disease to the vulva were noted.

### Surgical treatment options offered (Table 2)

**Table 2:** Surgical treatments offered to patients with vulvar carcinoma

*Surgical treatment*	*Frequency*	*Percentage (%)*
Wide local excision	1	9.1
Simple vulvectomy	1	9.1
Hemi - vulvectomy	2	18.2
Radical vulvectomy	6	54.5
Pelvic exenteration	0	0.0
Excision of the Bartholin's	1	9.1

**Total**	**11**	100.0%

The surgical treatment for the various stages of vulvar carcinomas offered to the patients ranged from wide local excision to radical vulvectomy. One patient had wide local excision for vulvar carcinoma, and 2(18.2%) of the women had hemi-vulvectomy. One woman had a simple vulvectomy. Six of the patients had radical vulvectomy as surgical treatment for vulvar carcinoma. One patient had excision of the Bartholin's gland.

### Complications of treatment (Table 3)

**Table 3:** Complications of surgery

*Complication*	*Frequency*	*Percentage (%)*
Anesthetic	1	9.1
Hemorrhage	2	18.2
Hematoma	1	9.1
Infection	1	9.1
Necrosis of skin flaps	0	0.0
Deep venous thrombosis	0	0.0
Pulmonary embolism	0	0.0
Lymphocyst formation	2	18.2
Chronic lymphedema	1	9.1
Urinary/fecal incontinence	0	0.0
Introital stenosis	1	18.2

***Total***	9	**100.0%**

* Some patients had more than one complication.

Some of the patients that had treatment for vulvar carcinoma developed complications from the treatment. One patient developed an anesthetic complication (Mendelson's syndrome). Two (18.2%) of the patients developed lymphocysts and 1 woman had chronic lymphedema as a complication of surgical treatment. One of the patients developed secondary wound infection.

### Stage at presentation

Of the women who had vulvar carcinoma, 1(9.1%) presented with stage IB, 1(9.1%) with stage II, 5(45.4%) with stage III and 4(36.4%) with stage IV disease.

## Discussion

Vulvar cancers constituted 1.27% of all gynecological admissions from this study. This is low when compared to a study in Ghana, where vulvar carcinomas constituted 2.21% of all gynecological malignancies [7]. The mean age for vulvar carcinoma from this study - 61.2 years, is within the normal age range for vulvar carcinomas when one considers the fact that even early invasive carcinoma of the vulva is known to be most common in the late fifties and early sixties. Indeed, in the series from Zimbabwe, all but one of the 31 patients with vulvar carcinoma were older than 55 years [8]. It may be mentioned, however, that a mean age of 43.8 years was reported from Jamaica about forty years ago [9] and two patients with vulvar carcinoma in the study done in Nigeria 20 years ago were in the 40-49 years group [6].

Women in Nnewi, Nigeria with vulvar cancer often present late for treatment, perhaps because some of them are not well informed about the disease and the need to present early. The women in this study were from the low socio-economic group and had no formal education. This might have been the reason why they presented late for treatment with advanced vulvar cancer. As was noted, 9 of the 11 women presented for the first time with at least, stage III disease. Again, even when they present for treatment, the very elderly ones are usually reluctant to be physically examined. These are all challenges to diagnosis and treatment of this disease in Nnewi, Nigeria.

The majority of the women in this study presented with stage III vulvar carcinoma (45.4%). Some clinicians may fail to recognize vulvar carcinoma when patients present to the hospital in very early stages because of the vague nature of the symptoms and rarity of vulvar cancer. It is possible that even when some of these women present early enough to physicians, the correct diagnosis may not made on time. Hence, they may progress to late stages of the disease before the correct diagnosis is made. It is of note here that the staging system used for vulvar carcinoma in this study was the FIGO staging system of 1988, (since all the cases had been diagnosed and treated before the new 2009 FIGO staging system was officially released). A new FIGO system for vulvar carcinoma was just adopted by the FIGO committee on gynecologic oncology in 2009 [10]. The new FIGO system of staging vulvar carcinomas emphasized the importance of lymph node metastasis in the staging, treatment and prognosis of vulvar carcinomas.

The commonest symptom of vulvar carcinoma from this study was pruritus vulvae (seen in 26.4% of the patients). Other symptoms of vulvar carcinoma include vulvar bleeding, vulvar swelling, ulceration, pain or burning sensation and vaginal/vulvar discharge [11,12]. The patients studied presented with chronic vulvar itching, vulvar bleeding, discharge, vulvar swelling and symptoms of lower urinary tract infection. Vulvar carcinoma is usually asymptomatic in up to 5% of cases, where it may be detected histologically in association with vulvar intra-epithelial Neoplasia (VIN) or carcinoma of the cervix or anus [7]. On examination, the typical appearance of vulval cancer is an ulcer with raised or rolled edges [11,12] as was seen in the patients in this study with advanced disease.

Metastatic disease to the regional inguino-femoral lymph nodes is the most important factor determining survival. The depth of invasion of the primary tumor is the most important factor for predicting nodal involvement. When the depth of invasion is between 1 and 2 mm, the incidence of positive nodes is 8% [2,11]. This rises to 30% if the depth of invasion is between 3 and 5 mm [3,11]. Lymphadenectomy was not done in the patient that presented with basal cell carcinoma of the vulva because the lymph node biopsy result was negative. Also, it is known that basal cell vulvar carcinoma rarely metastasize to the regional lymph nodes. Secondly, the morbidity associated with lymph node dissection may be high. These were justifications for treating the patient that presented with basal cell carcinoma with wide local excision without lymph node dissection. However, in patients with advanced vulvar carcinoma, radical vulvectomy and bilateral lymphadenectomy was done.

Wide local excision involves using a circumferential incision to remove the cancer and some of the normal tissue around the cancer. This was the treatment of choice offered to the patient that presented with basal cell carcinoma. The patient was placed in the lithotomy position, cleaned with chlorhexidine solution and draped. Local infiltration was done using 10 mls of 1% lignocaine around the area the ulcer was located on the upper portion of the right labia majora. Vulval skin excision lines were marked, and a 2 cm margin of normal skin surrounding the lesion was taken with the incision. The raw surfaces were closed. The post-operative condition was satisfactory.

Vulvar biopsy is usually carried out in the clinic under local anesthesia. In lesions that are 2 cm or less, a wide local excision biopsy is appropriate but should include a surrounding 2 cm zone of normal tissue [11,12]. In large ulcerated lesions, biopsies should be taken from the edge of the tumor and should include some normal skin [4]. Diagnosis of the tumor in these patients was based upon a representative biopsy that included an area where there was a transition of normal to malignant tissue. Biopsy specimens were taken from the vulva in all the 11 women that presented with vulvar cancer for histological diagnosis prior to the definitive treatment they had. Biopsies collected from these women were of sufficient size to allow differentiation between superficially invasive and frankly invasive vulvar tumors. Vulvar biopsies are very important prior to treatment because they help to determine the extent of disease spread, as well as the histological type of the vulvar cancer [12-15]. Occasionally, an alternative strategy might be considered. In certain situations where the clinical diagnosis is apparent and the patient is very symptomatic, like in heavy bleeding and or in severe pain, definitive surgery for the vulvar lesion may be performed. However, in such situations, biopsy with frozen sections is recommended prior to proceeding with any radical procedure. There was no need to do frozen sections on any of the vulvar specimens collected in this study because the definitive diagnoses of vulvar cancer in these patients were already made histologically prior to surgery.

The vulvar cancer patients that were managed in this study were generally elderly women. These patients often have co-morbidities, so a pre-operative anesthetic assessment can be invaluable. The women that presented with vulvar cancer in this study had pre-operative assessment by the anesthetists' a day prior to surgery to determine their fitness for surgery. Pre-operative investigations that were done for them included full blood count, serum biochemistry, chest X-ray and electrocardiogram. All these investigations were done for the patients because majority of them were elderly. One of the women developed aspiration pneumonitis (Mendelson's syndrome) as a complication of general anesthesia. This was however, managed in the intensive care unit and the patient recovered fully.

Radical vulvectomy and bilateral inguinal lymphadenectomy was done for 6 of the women that presented with advanced disease. We inserted Foley catheter into the bladder for continuous drainage after surgery. The wounds were assessed during surgery to determine whether they could be closed without tension by mobilizing adjacent tissue. All our radical surgeries were closed satisfactorily since we were able to mobilize the tissues around the vulva. Radical vulvectomy was associated with physical morbidity in 4 patients. Two of the patients developed lymphocysts. The lymphocysts were treated by marsupialization. The patient with lymphedema was treated with external compression stockings. She was also advised about rest, exercise, skin care and massage.

Extensive excisions can create difficulties with primary wound closure, leading to wound dehiscence, extensive scarring and disfigurement, discomfort, depression and loss of sexual function [11]. Hence, in the 6 patients that had radical vulvectomy, frank and open discussions with the patients and their families was essential at every stage of treatment to establish the patients' wishes as well as to determine treatment intent in the light of the likely prognosis.

Butterfly incisions have been used for the treatment of vulvar carcinoma in the past. In 1981, Hacker et al [12] showed that, for patients with stage I and II disease, survival using a triple incision technique was equal to the butterfly incision with reduced morbidity. Surgery through the triple incision is now the gold standard procedure. These findings have been confirmed by other studies [12]. In stage IA tumors, the risk of nodal metastases is virtually zero. Therefore, the tumor can be removed by wide local excision with a 2 cm margin of normal tissue. Groin node dissection should be avoided if there is less than 1 mm of invasion. For early vulvar cancers (stage IB and II), a wide local excision down to the perineal fascia is acceptable. However, if the disease is multifocal, a wider excision will be required. It should be noted that the triple incision technique is used to accommodate inguinal lymph node removal.

The use of pre-operative radiotherapy and chemotherapy may shrink a vulvar tumor to allow less destructive surgery, in particular, preservation of sphincters and avoidance of stomas in stages III and IV carcinomas of the vulva, especially of the squamous type. These were not done in the patients that presented with stage III and IV diseases because the tumors were excised without much difficulty and the sphincters were avoided satisfactorily. These patients were sent for adjuvant radiotherapy after surgery. One of the women had a left hemi-vulvectomy because the mass was completely limited to the left vulva.

It must also be noted that following surgical treatment of vulvar carcinoma in this study, the vulvar tissues excised were sent for histology. It was not possible to do human papilloma virus (HPV) testing on the tissues because the facilities for testing vulvar tissues for HPV are not readily available in Nigeria. Only very few teaching hospitals in Nigeria, like University College Hospital, Ibadan, Nigeria; Ahmadu Bello University Teaching hospital, Zaria, Nigeria; University of Lagos Teaching Hospital, Nigeria and National Hospital, Abuja, Nigeria have the facilities to test for HPV. Even in these centers, there are very few histopathologists who carry out these procedures.

This study was conducted in one of the tertiary referral centers in south-eastern Nigeria. One limitation of this study was that it was conducted in one tertiary center in Nigeria. However, the fact that this tertiary center is among the biggest referral centre in south-eastern Nigeria makes it a worthwhile study. Patients with gynecological cancer are referred to the Obstetrics and Gynaecology department of the hospital from all parts of the country. The hospital receives and manages complicated cases mis-managed by traditional healers from all parts of Nigeria. Up till 2010, there are no functional facilities for radiation therapy in south-eastern Nigeria. Women with advanced vulvar carcinoma who need radiotherapy are referred to one of any three centers in the country where they can receive treatment - the south-western part of Nigeria (Lagos or Ibadan) or the northern part of the country (Abuja or Zaria). However, majority of them do not go for this treatment because they cannot afford it.

## Conclusion

Vulvar carcinoma is a rare gynecologic malignancy. The malignancy is amenable to treatment if patients present early enough. Currently, most radiotherapy machines in Nigeria are non functional. Since most of the patients with vulvar carcinoma present in advanced stages of the disease, management becomes difficult. Hence, reducing morbidity associated with treatment without compromising on cure rates remains a challenge.

## Conflict/Disclosure of interests

The authors declare that they have no competing interests.

## Authors' contributions

AE, LAI and JA were all involved in the study conception and design, acquisition of data, analysis and interpretation of data, drafting of manuscript and the critical revision of the manuscript. All authors read and approved the final manuscript.

## References

[B1] Mohammed A Ahmed SA Oluwole OP Avidine S Carcinomas of the Genital tract at ABUTH, Zaria Annals of African Medicine 2006 5 2 93 96

[B2] Siddiqui N The management of vulval cancer Curr Obstet Gynaecol 2002 12 97 103 10.1054/cuog.2001.0241

[B3] RCOG Publications Management of vulval cancer 2006 London, Royal College of Obstetricians and Gynaecologists

[B4] De-Cherney AH Goodwin TM Nathan L Laufer N Vulval Cancer Current diagnosis & treatment Obstetrics & Gynecology 2007 10 McGraw-Hill 822 7

[B5] Luesley D The management of vulval cancer and related conditions Obstet Gynaecol 1999 1 London 7 12

[B6] Briggs ND Katchy KC Pattern of primary gynaecological malignancies as seen in a tertiary hospital situated in the Rivers State of Nigeria Int J Gynecol Obstet 1990 31 157 61 10.1016/0020-7292(90)90714-V 1968863

[B7] Nkyekyer K Pattern of gynecological cancers in Ghana East Afr Med J 2000 77 10 534 8 12862120 10.4314/eamj.v77i10.46708

[B8] Kasule J The pattern of gynaecological malignancy in Zimbabwe East Afr Med J 1989 66 393 9 2791944

[B9] Hay DM Cole FM Primary invasive carcinoma of the vulva in Jamaica J Obstet Gynaecol Brit Commwlth 1969 76 821 830 5823682

[B10] Perecolli S Revised FIGO staging for carcinoma of the vulva, cervix and endometrium Int J Gynaecol Obstet 2009 105 2 103 4 10.1016/j.ijgo.2009.02.012 19367689

[B11] Ansink A Van der velden J Collingwood M Surgical interventions for early squamous cell carcinoma of the vulva CD002036 10796849 10.1002/14651858.CD002036 PMC8078494

[B12] Hacker NF Leuchter RS Bereck JS Castaldo TW Lagasse LD Radical vulvectomy and bilateral inguinal lymphadenectomy through separate groin incisions Obstet Gynecol 1981 58 574 9 7301232

[B13] Grimshaw RN Murdoch JB Monaghan JM Radical vulvectomy and bilateral inguino-femoral lymphadenectomy through separate incisions: experience with 100 cases Intl J Gynecol Cancer 1993 3 18 23 10.1046/j.1525-1438.1993.03010018.x 11578318

[B14] Hacker NF Van der velden J Conservative management of early vulvar cancer Cancer 1993 71 1673 7 8431905 10.1002/cncr.2820710436

[B15] Dennerstein G Scurry J Brenan J Allen D Marin M-G The vulva and vagina A textbook of Gynaecology for post-graduates 2007 315 331

